# Windkessel model of hemodynamic state supported by a pulsatile ventricular assist device in premature ventricle contraction

**DOI:** 10.1186/s12938-018-0440-5

**Published:** 2018-02-02

**Authors:** Keun Her, Joon Yeong Kim, Ki Moo Lim, Seong Wook Choi

**Affiliations:** 1 Department of Cardiovascular and Thoracic Surgery, Soonchunhyang University Hospital, Bucheon-si, Republic of Korea; 2 Program of Mechanical and Biomedical Engineering, College of Engineering, Kangwon National University, Chuncheon-si, Republic of Korea; 3 Department of Medical IT Convergence Engineering, Kumoh National Institute of Technology, Gumi, Republic of Korea

**Keywords:** Windkessel model, Pulsatile ventricular assist device, Counter-pulsation control, Phase-locked loop, Arrhythmia

## Abstract

**Background:**

Counter-pulsation control (CPC) by ventricular assist devices (VADs) is believed to reduce cardiac load and increase coronary perfusion. However, patients with VADs have a higher risk of arrhythmia, which may cause the CPC to fail. Consequently, CPC has not been applied by VADs in clinical practice. The phase-locked loop (PLL) algorithm for CPC is readily implemented in VADs; however, it requires a normal, consistent heartbeat for adequate performance. When an arrhythmia occurs, the algorithm maintains a constant pumping rate despite the unstable heartbeat. Therefore, to apply the PLL algorithm to CPC, the hemodynamic effects of abnormal heartbeats must be analyzed.

**Objectives:**

This study sought to predict the hemodynamic effects in patients undergoing CPC using VADs, based on electrocardiogram (ECG) data, including a wide range of heart rate (HR) changes caused by premature ventricular contraction (PVC) or other reasons.

**Methods:**

A four-element Windkessel hemodynamic model was used to reproduce the patient’s aortic blood pressure in this study. ECG data from 15 patients with severe congestive heart failure were used to assess the effect of the CPC on the patients’ hemodynamic state. The input and output flow characteristics of the pulsatile VAD (LibraHeart I, Cervika, Korea) were measured using an ultrasound blood flow meter (TS410, Transonic, USA), with the aortic pressure maintained at 80–120 mmHg. All other patient conditions were also reproduced.

**Results:**

In patients with PVCs or normal heartbeats, CPC controlled by a VAD reduced the cardiac load by 20 and 40%, respectively. When the HR was greater for other reasons, such as sinus tachycardia, simultaneous ejection from the heart and VAD was observed; however, the cardiac load was not increased by rapid cardiac contractions resulting from decreased left ventricle volume. These data suggest that the PLL algorithm reduces the cardiac load and maintains consistent hemodynamic changes.

## Background

Use of a ventricular assist device (VAD) is the most effective way to improve the survival rates of patients with end-stage heart failure [1–5]. However, to avoid additional heart problems or thrombus, new approaches such as pulsatile flow and counter-pulsation control (CPC) are required [5, 6]. Although CPC is expected to increase cardiovascular circulation and reduce cardiac load, it has not been implemented clinically because of the possible adverse effects on the heart in the event of CPC failure [7–9]. Patients with VADs are at a higher risk of developing arrhythmia, which can induce CPC failure [10, 11]. Therefore, prior to engaging in CPC, it should be confirmed that failure of the VAD will not cause the patient’s hemodynamic state or cardiac load to worsen.

Multiple studies of CPC using VADs have assumed normal HRs without considering the possible occurrence of arrhythmias. Lim et al. reported that CPC increased coronary perfusion and decreased cardiac load [7]. Koshimoto et al. assessed such factors using pulsatile rotary VADs in animal studies [8, 9]. Some CPC algorithms have considered tachycardia and bradycardia; however, this approach has not been tested clinically or in animals due to the difficulties associated with inducing arrhythmias [12]. Clinical studies involving CPC are complicated despite the increasing use of VAD applications. This is because many reports have suggested that VADs should not be used for patients with arrhythmias [13–15]. Moreover, most VADs used to date did not have a CPC function [12, 16]. Intra-aortic balloon pumps (IABPs) have successfully engaged in CPC and beneficially affected heart treatment [17, 18]. However, in cases involving VADs, it is difficult to apply the CPC algorithm for IABPs. IABPs inject helium gas through a catheter into the balloon in the aorta rapidly, whereas the outflow pulse of a VAD as it passes through the blood stream is slow [9, 18, 19]. Therefore, the CPC algorithm of VADs should predict future heartbeats based on prior heartbeats, and preemptively provide subsequent pulses [12].

The LibraHeart I (Cervika, Korea) is a pulsatile VAD that analyzes prior heart beats and initiates its next pulse flow to keep the time interval between the pulses of the patient’s heart and the VAD constant according to the phase locked loop (PLL) algorithm [6]. The PLL is an algorithm that determines the pumping rate of a VAD whose output pulses are related to the QRS complex of the patient’s electrocardiogram (ECG) [20]. However, the PLL algorithm for a VAD can fail at CPC when arrhythmias occur. Therefore, the likelihood of CPC failure, and the effects it would have on cardiac load and the hemodynamic state, should be investigated before determining whether to implement CPC using VADs. This study analyzed ECG data from 15 patients with congestive heart failure and observed 35 arrhythmias, including premature ventricular contractions (PVCs) and sinus tachycardia (ST), in those ECGs [21].

The hemodynamic model was based on the four-element Windkessel model, which reproduces patient blood pressure and blood flow data [22, 23]. Because the conventional Windkessel model does not consider the reflection wave in the aorta, the actual blood pressure and blood flow data of a patient cannot be reproduced [24]; whereas the four-element model considers the reflection wave and reproduces the same aortic pressure and blood flow as measured in the patient [25]. The four-element model has been used to evaluate the accuracy of medical devices that analyze blood pressure and blood flow [22].

The purpose of this study was to predict the effects of a VAD in CPC mode on the cardiac and hemodynamic states using a four-element Windkessel model that includes the data for a VAD and arrhythmias that induced CPC failure.

## Methods

### Counter pulsation control by the ventricular assist device using the phase-locked loop method

The conventional method of determining when to initiate VADs following heartbeat detection misses the appropriate counter-pulsation point. If the delay from the time of heart rate (HR) detection to actuator operation is not reduced to within 0.1 s [9], a normal heartbeat of over 70 bpm can be missed. Following heartbeat detection, delay occurs due to the time required to establish the pulse flow by increasing the actuator speed, thereby generating the blood pressure pulse at the outlet of the VAD and delivering the pulse to the aortic valve through vessels and blood [18]. Comparatively, an IABP successfully applies CPC and reduces delays by injecting helium gas through a stiff, narrow catheter to a balloon located near the heart [19]. To overcome the slow response of a pulsatile VAD, the controller should predict the starting point by analyzing the regularity of the heartbeat and increasing motor speed prior to detecting the following beat [6, 12].

**Fig. 1:**
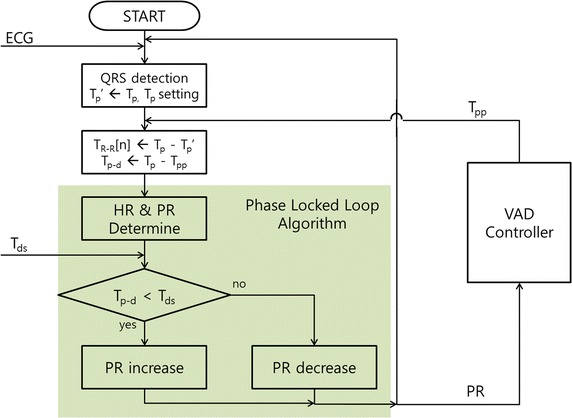
Phase-locked loop (PLL) algorithm for counter-pulsation control (CPC)

A pulsatile VAD (LibraHeart I, Cervika, Korea) uses a PLL algorithm to determine the starting point for pulse generation (Fig. 1) [6, 26]. The VAD controller sets the pumping rate (PR) of the VAD to equal the patient’s HR. The HR is determined by measuring the heartbeat time (T_p_) and calculating the beating period (T_R-R_). The heartbeat time (T_p_) is determined from ECG data using the Tompkins method [27]. The HR is calculated as the average reciprocal of the normal T_R-R_ over a 10-s period. The means and standard deviations of all T_R-R_ values during the 10-s period are calculated, and T_R-R_ values that fall within the range of the mean and standard deviation are regarded as normal values. Since arrhythmias, including PVC, affect the T_R-R_ average and HR, if the PR is affected by outlying data, the CPC of a VAD may have constant errors for even normal heartbeats. As the onset of pulse generation is determined prior to the heartbeat being detected, the delay (T_p-d_) between the heartbeat and the VAD pulse is calculated immediately following T_p_ detection. When the T_p-d_ differs from the predetermined optimal value (T_ds_), the ensuing VAD pulse is adjusted temporarily to bring T_ds_ to within 5% of the average T_R-R_. T_ds_ was determined such that the ratio of T_p-d_ to T_R-R_ was 45%.

When an arrhythmia occurred, T_R-R_ changed temporarily from 30 to 170%, which caused temporary CPC failure. In most cases, however, the VAD slowly adjusted its PR to the average HR according to the PLL algorithm, and it seemed to maintain a constant PR as opposed to changing the PR of the IABP for each heartbeat.

### Hemodynamic model of the left ventricle and aorta

The hemodynamic changes were investigated using the four-element Windkessel model. The conventional Windkessel model shows the relationship of diastolic and systolic blood pressure and blood flow [24]. However, it does not reproduce the actual blood pressure and blood flow waveform, so that a more sophisticated model, such as a four-element Windkessel model, is necessary [22, 25]. The four elements of the Windkessel model are the aortic compliance, aortic impedance (resistance and inductance of the aorta), resistance of the peripheral arteries, and compliance of the peripheral arteries. These elements are necessary to reproduce the reflection wave, actual blood pressure, and blood flow information of an actual patient [25]. The values used in previous studies were used again to reproduce the blood pressure and blood flow data, as shown in Table 1 [22].

**Table 1:** Parameters related to reflection wave in the 4-element Windkessel model

Symbol	Values	Units	Meaning of parameter
C_Aorta_	0.15	cc/mmHg	Compliance of the aorta
Z_Aorta_			
L_Aorta_	0.0015	cc/s^2^	Impedance of the aorta composed of inertia and resistance of blood flow
R_Aorta_	0.08	mmHg s/cc	
C_PA_	1.5	cc/mmHg	Compliance of the peripheral arteries
R_PA_	1.35	mmHg s/cc	Resistance of the peripheral vessels

To compare the characteristics of the cardiac load in the four-element Windkessel model and to analyze the effect of the VAD, the compliance of the LV, the resistance of the mitral and aortic valves, and the LA pressure were determined, as shown in Table 2.

**Table 2:** Auxiliary parameters used to estimate the cardiac load supported by the VAD

Symbol	Values	Units	Meaning of parameter
C_LV_	12.5	cc/mmHg	Compliance of the LV during LV relaxation
	0.66–12.5		Compliance of the LV during LV contraction
R_AV_	0.002	mmHg s/cc	Resistance of the aortic valve when it opens
	1000		Resistance of the aortic valve when it closes
R_MV_	0.01	mmHg s/cc	Resistance of the mitral valve when it opens
	1000		Resistance of the mitral valve when it closes
P_LA_	10	mmHg	Blood pressure in the left atrium
VAD	2 (average)	L/min	Blood flow through the VAD

**Fig. 2:**
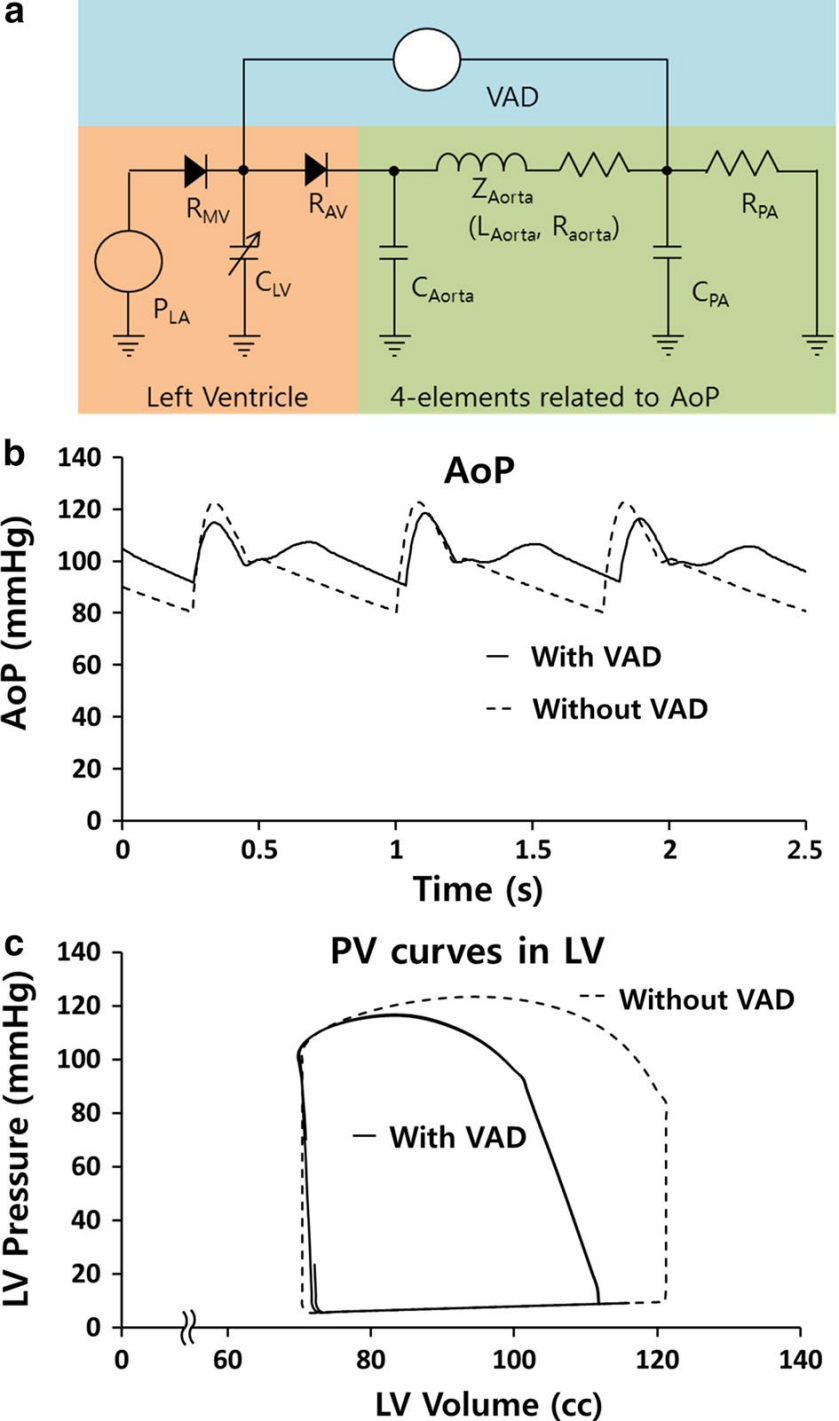
**a** 4-element Windkessel model, including the ventricular assist device (VAD), **b** aortic blood pressure (AoP), and **c** pressure–volume curves before and during VAD perfusion

The C_LV_ is the ratio of volume to pressure in the LV when the internal pressure changes from 5 to 120 mmHg and the blood volume changes from 65 to 120 cc (Fig. 2c) [24]. The values of R_AV_ and R_MV_ are the resistance of valve opening and closing according to the direction of the blood flow [24]. P_LA_ is the pressure of the left atrium and was determined to be 10 mmHg, which is the normal left atrium pressure.

**Fig. 3:**
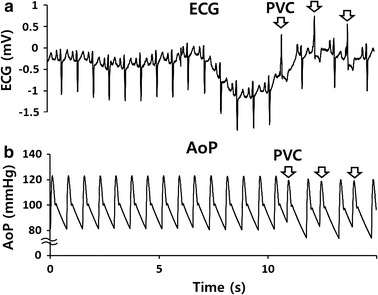
**a** Representative electrocardiogram (ECG) data showing temporary premature ventricle contraction (PVC) obtained from a congestive heart failure patient, and **b** the patient’s AoP, reproduced by the Windkessel model

In the absence of the VAD, HRs of 70 bpm resulted in an arterial pressure of 80–120 mmHg and an average cardiac output of 4 L/min, as shown in Fig. 2b. With the exception of the VAD connection, the Windkessel model was based on patient data, with the reflection wave being adjusted to reproduce the same waveform as for the patient’s actual aortic blood pressure (AoP) [22, 23]. When a pulsatile VAD that implements the PLL algorithm is applied to the Windkessel model, the systolic (diastolic) BP should decrease (increase). This should reduce the LV volume, and the decrease in LV output and LV pressure will result in a decrease in load, which is similar to the results of previous animal experiments [6]. The Windkessel model initiated LV contractions at the QRS wave on the ECG, as shown in Fig. 3. As a result, when an arrhythmia occurred on the ECG, the changes in HR induced changes in the blood pressure and blood flow in the LV and the aorta of the Windkessel model.

ECG data were obtained from 15 patients (11 men, aged 22–71 years; 4 women, aged 54–63 years) with severe congestive heart failure (NYHA class 3–4) at a sampling rate of 250 per second. The data included 1260 heart beats and 28 arrhythmias [21]. Nine patients had tachycardia with a rapid HR over 100 bpm. The primary arrhythmias were PVCs (Fig. 3a), which animal studies suggest are more likely to occur when using a pulsatile VAD [6]. Although the contraction force of the left ventricle was set to be constant, the Windkessel model predicted that the AoP would decrease during PVC (Fig. 3b), as shown in animal studies. In total, 25 PVCs were analyzed among 15 patients and were found to increase the HR by 15.5–34.5%. LV contractions following a PVC occurred regularly, so the T_R-R_ following PVCs was temporarily extended, changing the hemodynamic states in the model. Such hemodynamic changes at PVC and at the next beat were observed and compared as the LV stroke volume fluctuated. The HR changes caused by PVCs were applied to two Windkessel models, one with a VAD and one without a VAD. Three episodes of ST occurred in two patients and resulted in simultaneous ejection of the heart and VAD. The effect of co-pulsations was compared with the results for normal counter-pulsation. In addition, the relationship between temporary heart rate changes and the cardiac load was investigated by applying HR changes over a wide range to the model.

**Fig. 4:**
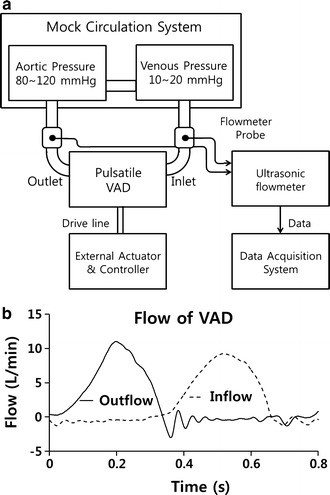
**a** Experimental setup for the flow measurement of the pulsatile VAD, and **b** the inflow and outflow waveform of the VAD

The inflow and outflow data for the VAD (LibraHeart I, Cervika, Korea) were measured using an ultrasonic blood flow meter (TS410, Transonic, USA) using a mock circulation system that reproduces the patient’s hemodynamic conditions [26]. As shown in Fig. 4a, the mock circulation system consists of a compliance chamber that maintains the afterload of the VAD from 80 to 100 mmHg and an open chamber that maintains the preload of the VAD at 10 mmHg as the AoP of the Windkessel model and the patient. The pulse volume of the VAD was fixed at 29 cc, so that the outflow of the VAD reached 2 L/min, which is 50% of the normal body perfusion rate when the pulse rate was 70 bpm without a VAD (Fig. 4b). The model assumes that the inlet and outlet of the VAD are connected to the LV and the descending aorta, as shown in Fig. 2a.

## Results

**Fig. 5:**
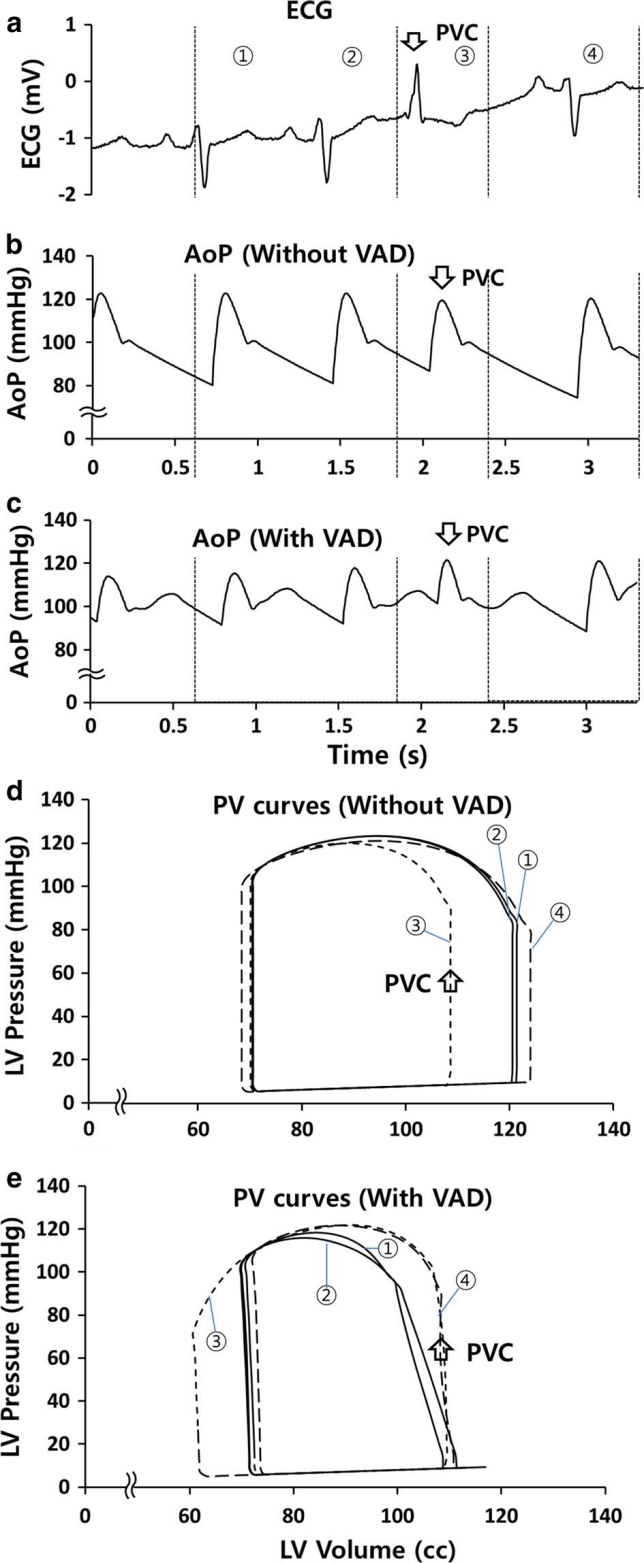
Representative curves for **a** patient ECGs, **b** AoP without VAD perfusion, **c** AoP with VAD perfusion, **d** pressure–volume (PV) curves of LV without VAD, and **e** PV curves of LV with VAD. At first, the patient’s heart beat was normal (①, ②). However, when PVC occurred, the heart rate increased abruptly (③) and then immedicately decreased (④)

PVCs stopped the blood flow from the left atrium to the LV and reduced the maximum volume within the ventricle during a heartbeat, as shown in Fig. 5. In Fig. 5, ① and ② are representative of normal ventricular contractions before PVCs, while ③ is representative of a case when a PVC occurs. The heartbeat immediately following the PVC occurred at the normal time point, but the volume of the ventricle increased beyond the normal value due to the increased time between the PVC and the subsequent heartbeat (Fig. 5d). The PV curve in Fig. 5d shows the changes in ventricular pressure and volume caused by PVCs. Changes in the area of the closed curve reveal that the cardiac load differed between PVC and normal heartbeats.

The volume of the ventricle was reduced by the inflow to the VAD (Fig. 5e). When PVC occurred and the ratio of T_p-d_/T_R-R_ deviated significantly from the target value (45%), the cardiac output and the output of the VAD did not occur simultaneously, because the blood flow output period of the VAD was too short, so the VAD output finished before the start of the following PVC. As shown in Fig. 5b, c, the diastolic AoP increased due to the output of the VAD, while the systolic pressure decreased due to decreases in the cardiac output caused by the flow from the LV to the VAD. The VAD pulses caused the area of the PV curve to decrease (Fig. 5e). Even when the PVC was generated, the VAD reduced the volume of the ventricle, which subsequently reverted to its prior size following the PVC.

**Fig. 6:**
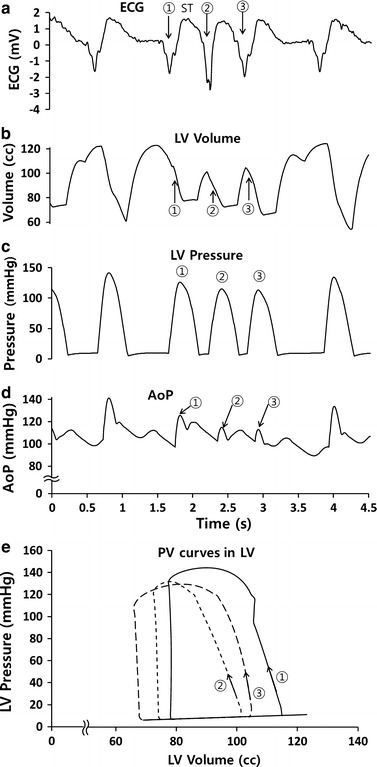
Representations of **a** patient ECGs, **b** LV volumes, **c** LV pressures, **d** AoP and **e** PV curves during normal (①) and subsequent abnormal heart beat caused by sinus tachycardia (②, ③)

When ST occurred, successive rapid heartbeats induced co-pulsation, as shown in Fig. 6a. However, the change in LV volume remained small because the VAD pumped blood from the LV to the aorta continuously (Fig. 6b). The systolic BP did not increase, but decreased (Fig. 6d). After finishing ST, the diastolic BP decreased slightly as the heart rate decreased. The PV curves showed that CPC failure did not increase the cardiac load.

**Fig. 7:**
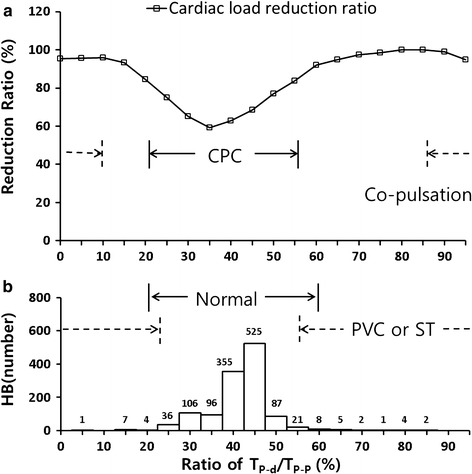
**a** Predicted cardiac load reduction ratio according to the ratio of T_p-d_ to T_R-R_, and **b** histogram of the ratios of T_p-d_ to T_R-R_ for observed patients’ heartbeats

As shown in Fig. 7b, a total of 1260 heart beats occurred in 15 patients, including 25 PVCs and three episodes of ST (10 beats). Considering the 1225 beats excluding PVCs and ST, the ratio of T_p-d_ to T_R-R_ also changed from 20 to 60% due to unknown HR changes and tachycardia (> 100 bpm). When the ratio of T_p-d_ to T_R-R_ was within 20–60%, the cardiac load was expected to decrease by 20–40% (Fig. 7a).

## Discussion and conclusion

Although PVCs are not dangerous arrhythmias, their frequency affects the occurrence of dangerous arrhythmias such as ventricular fibrillation [28, 29]. PVC data enable analysis of the effect of various types of arrhythmia, because PVC shows fast and slow heart rate changes within a short period. In animal studies using pigs and a PLL-controlled VAD, CPC by the VAD induced clear changes and the systolic blood pressure decreased by 23%, while the diastolic BP increased by 25% [6]; this resulted from the different characteristics of animals compared with humans and edema that was observed at thoracotomy. In vivo, PVCs occurred, when the VAD operated not in CPC mode, but in asynchronous mode. Changes in ventricular load caused by VADs are thought to cause PVCs [30]. As a result, the animal studies did not show the effects of CPC on the hemodynamic state during arrhythmias, because arrhythmias did not occur in the presence of CPC. As the causes of PVCs include alcohol consumption and stress [28], PVC can still occur, even when a VAD imparts CPC; however, they are difficult to detect due to their intermittency. Thus, PVCs can be the prevailing arrhythmia, despite all efforts to avoid the use of VADs in arrhythmia patients.

The results of this study were obtained from simulations; however, the input and output blood flow data of the VAD were measured under the same conditions based on patients and in vitro [26]. The ECG data were measured from patients who required the application of a VAD [21]. The Windkessel model was designed using accepted mathematical and physical formulas relating to hemodynamic states [22]. The parameters in the model, including blood pressure, cardiac output, and systemic vascular resistance, were identical to those in another study and were measured in patients [22, 25]. Since the model includes the AoP waveform, which is similar to that measured in patients, the model is considered to include hemodynamic data [23]. Therefore, the results of this study are expected to be realistic for patients with similar hemodynamic characteristics.

The model used in this study predicted the effects of the VAD on events that were difficult to reproduce in clinical or animal experiments. However, a limitation of this study is that predictions using other input conditions require a substantial volume of data to be collected beforehand. Because the cardiovascular characteristics of patients vary due to multiple factors, including the influence of sympathetic and parasympathetic nervous systems and the patient’s movement and posture, predictions must encompass a wide range of conditions. As the technologies for acquiring data become more encompassing, the limitations of numerical simulation will be minimized [31, 32].

Previous studies of CPC were designed to detect heart rhythms and to maintain the VAD at constant intervals [9, 12]. However, in those studies, the VAD was expected to alter the blood flow irregularly, when irregular heartbeats occurred [33]. It is difficult to predict the outcome of CPC by a VAD under arrhythmia conditions, given that the algorithm accounts for complex scenarios to avoid co-pulsation [12, 19]. Because the Novacor left ventricular assist system (LVAS) (Novacor, Oakland, CA, USA) also has a PLL algorithm, our results should be useful for predicting the hemodynamic effects of the device during an arrhythmia [34].

In this study, the PLL algorithm showed a 97.3% success rate at CPC when tachycardia and arrhythmias appeared in patients and it could reduce the heart load. It predicted that CPC and temporary CPC failure would not induce an increased cardiac load, even when co-pulsation occurred.
